# Allochthonous and Autochthonous Halothermotolerant Bioanodes From Hypersaline Sediment and Textile Wastewater: A Promising Microbial Electrochemical Process for Energy Recovery Coupled With Real Textile Wastewater Treatment

**DOI:** 10.3389/fbioe.2020.609446

**Published:** 2020-12-17

**Authors:** Refka Askri, Benjamin Erable, Luc Etcheverry, Sirine Saadaoui, Mohamed Neifar, Ameur Cherif, Habib Chouchane

**Affiliations:** ^1^Univ. Manouba, ISBST, BVBGR-LR11ES31, Biotechpole Sidi Thabet, Ariana, Tunisia; ^2^Faculté des Sciences de Tunis, Université de Tunis El Manar, Tunis, Tunisia; ^3^Laboratoire de Génie Chimique, Université de Toulouse, CNRS, INPT, UPS, Toulouse, France

**Keywords:** halothermotolerant bioanodes, hypersaline sediment, textile wastewater, COD removal, energy recovery, autochthonous bacteria, allochthonous bacteria

## Abstract

The textile and clothing industry is the first manufacture sector in Tunisia in terms of employment and number of enterprises. It generates large volumes of textile dyeing wastewater (TDWW) containing high concentrations of saline, alkaline, and recalcitrant pollutants that could fuel tenacious and resilient electrochemically active microorganisms in bioanodes of bioelectrochemical systems. In this study, a designed hybrid bacterial halothermotolerant bioanode incorporating indigenous and exogenous bacteria from both hypersaline sediment of Chott El Djerid (HSCE) and TDWW is proposed for simultaneous treatment of real TDWW and anodic current generation under high salinity. For the proposed halothermotolerant bioanodes, electrical current production, chemical oxygen demand (COD) removal efficiency, and bacterial community dynamics were monitored. All the experiments of halothermotolerant bioanode formation have been conducted on 6 cm^2^ carbon felt electrodes polarized at −0.1 V/SCE and inoculated with 80% of TDWW and 20% of HSCE for 17 days at 45°C. A reproducible current production of about 12.5 ± 0.2 A/m^2^ and a total of 91 ± 3% of COD removal efficiency were experimentally validated. Metagenomic analysis demonstrated significant differences in bacterial diversity mainly at species level between anodic biofilms incorporating allochthonous and autochthonous bacteria and anodic biofilm containing only autochthonous bacteria as a control. Therefore, we concluded that these results provide for the first time a new noteworthy alternative for achieving treatment and recover energy, in the form of a high electric current, from real saline TDWW.

## Introduction

The textile and clothing industry has become one of the most important sectors of activity. Despite the use of high-tech equipment and modern technologies, it remains among the highest water consuming industries. As reported by the United States Environmental Protection Agency (USEPA), the production of 9,072 kg of finished textile per day requires about 36,000 L of water only for wet processing (Ghaly et al., 2014; Berkessa et al., 2020). Moreover, almost the water consumed generates large volumes of textile dyeing wastewater (TDWW). The TDWW contains more than 1.5 g/L of waste dye as the uptake of these dyes by textile fabrics is very poor, high salinity of about 5–6% NaCl and 5% of Na_2_SO_4_ and other chemicals, such as various acids, alkalis, sulfur, naphthol, surfactant-dispersing agents, formaldehyde-based dye fixing agents, hydrocarbon-based softeners, and heavy metals (Verma et al., 2012; Lin et al., 2015; Pazdzior et al., 2018). Most of these chemicals and products of their degradation, i.e., metabolites, are recalcitrant in nature and severely affect both aquatic and terrestrial life (Ben Mansour et al., 2012; Kant, 2012; Chandrakant et al., 2016). It is therefore well-established that these hazardous pollutants should be removed from TDWW by appropriate and effective methods prior to their release into the environment.

Different TDWW treatment methods and techniques have been implemented and evaluated over the past two decades. These methods involve (i) physical methods (coagulation–flocculation, adsorption, and filtration techniques), (ii) oxidation methods categorized as advanced oxidation processes (cavitation, photocatalytic oxidation, Fenton chemistry) and chemical oxidation using oxidizing agents (O_3_ and H_2_O_2_), and (iii) bioremediation methods (fungi, algae, bacteria) (Gosavi and Sharma, 2014; Yeap et al., 2014; Chandrakant et al., 2016; Chouchane et al., 2018; Jiang et al., 2018). For several reasons, such as eco-friendly, cost competitive, less sludge production, and giving non-hazardous metabolites or full mineralization (Hayat et al., 2015), the biological methods are qualified as the most sustainable method for wastewater treatment. However, biological methods cannot guarantee the achievement of required results of the TDWW, as some of the dye molecules or other chemical components are hazardous and/or recalcitrant to microorganism-driven degradation. The most appropriate method should be cost-effective, valuable in the degradation of resistant compounds, and produce safe and good quality effluent. There is therefore a great environmental and economical need to develop a processing technology that addresses these severe challenges. Tenacious, resistant, and exoelectrogenic microorganisms used in bioelectrochemical systems (BES) can perform the dual function of degrading pollutants and recovering in the form of electrical energy, the energy resulting from the oxidation of these pollutants. Indeed, previous studies reported that exoelectrogenic populations have favorably demonstrated an added value for the treatment of recalcitrant pollutants, such as azo dyes, petroleum hydrocarbon, and heavy metals (Adelaja et al., 2015; Choudhury et al., 2017; Monzon et al., 2017; Grattieri and Minteer, 2018; Vijay et al., 2018; Askri et al., 2019; Elabed et al., 2019a,b).

As already reported by Xie et al. (2011), organic and inorganic compounds in TDWW contain almost five times more energy than that consumed to treat it. Thus, it is hypothetically possible to extract electrical energy from the TDWW by applying well-adapted electrochemically active microorganisms capable of degrading recalcitrant pollutants, transmuting dangerous metabolites, and capturing an electronic flow that can be converted into electrical energy under high salinity. In this context, an earlier study by Askri et al. (2019) demonstrated the enrichment of efficient exoelectrogenic microorganisms from hypersaline sediment of Chott El Djerid (HSCE) able to produce a current density in the range of 7 A/m^2^ under combined high temperature and hypersaline conditions (temperature 45°C, salinity 165 g/L) using lactate as carbon source, i.e., anodic fuel. Halothermophilic microorganisms are thus suitable candidates for the treatment of the high saline wastewaters generated, for example, in the textile dyeing (2–10 g/L), seafood processing (8–20 g/L), tannery (40–80 g/L), and petroleum industries (few g/L to 300 g/L) (Shehab et al., 2017; Cherif et al., 2018; Askri et al., 2019).

The aim of this work was therefore to demonstrate, for the first time, proof of the feasibility of designating an efficient microbial halothermotolerant bioanodes from both hypersaline sediment (HSCE) and saline TDWW microbium able to treat textile wastewater and generating an electrical current collected *via* an electrode. At this stage of progress, the exploitation of the anodic current flow generated is not investigated at all. It is only quantified in terms of bioelectrochemical kinetics, from the acquisition of the J = f(E) curves. It is thus not at all a question here of developing a microbial fuel cell (MFC), a microbial electrolysis cell (MEC), or any other BES, judged too fluctuating and random to focus attention on the precisely targeted phenomenon, i.e., the formation of a bioanode without limitation caused by a limiting step of a larger and more complete BES process. On the contrary, bioanode formation was studied here in three-electrode electrochemical bioreactor installations to ensure well-controlled electroanalysis conditions, as explained in the review by Rimboud et al. (2014), who point out the fundamental basic principles and advantages of the three-electrode arrangement compared with MFC or other BES devices.

Once the potential for electrical current generation and TDWW treatment of the halothermotolerant bioanodes had been proven *via* electrochemical and analytical tools, contributions of autochthonous microorganisms from TDWW and allochthonous from HSCE were investigated through comparative metagenomic analyses of biofilms, HSCE, and TDWW.

## Materials and Methods

### Collection of Hypersaline Sediment and Textile Wastewater Samples

Hypersaline sediments used as inoculums were sampled from an extreme environment Chott El Djerid, located in the south of Tunisia (N 33°59′965″ and E 08°25′332″). Samples were a mixture of saturated water and sediment (2:1 vol.:vol.) collected from the surface with a conductivity higher than 200 mS cm^−1^ (Askri et al., 2019). The TDWW was collected from “Sitex” textile industry located in Monastir, Tunisia (N 35°39′06″ and E 10°53′03″). All samples were conserved in closed plastic bags and bottles at +4°C until experiments were started.

### General Condition of Three-Electrode Bioelectrochemical Cell Configuration and Operation

All experiments were conducted in a 500 ml glass three-electrode reactor containing 80% of real textile effluents and 20% of saline sediments. After the homogenization step, reactors were hermetically closed without any gas flow. A conventional three-electrode system [working electrode (WE), auxiliary electrode, and reference electrode] was implemented with a VSP multichannel potentiostat (Biologic SAS) equipped with EC lab software. The WE made of a porous carbon felt of 6 cm^2^ projected surface area was electrically connected to a platinum wire (1 mm diameter and 15 cm long) and polarized at −0.1 V/SCE. A platinum grid was used as the counter electrode (CE), and a saturated calomel reference electrode (+0.24 V/SHE) was located between the counter and the WEs. Cyclic voltammetry (CV) was performed *in situ* between −0.6 and 0.3 V/SCE at a scan rate of 1 mV.S^−1^.

### Electrochemical Data Processing

Chronoamperometric (CA) maximum current densities of the halothermotolerant biofilms were carried out as indicated in the previous study by Askri et al. (2019). CV was used to (i) validate the formation of an electroactive biofilm by comparing a control CV of the WE immersed in the TDWW with HSCE before starting the CA and a turnover CV at the end of the experiments and (ii) get access to I = f(E) of the bioanode that was shaped.

### COD Measurement

The chemical oxygen demand (COD) removal rate (%) corresponds to the percentage of the total organic matter eliminated in the wastewater. Therefore, COD change monitoring is a relevant parameter to evaluate the performance of the halothermotolerant bioanode. The monitoring was carried out according to the following equation:

$$\begin{array}{l} \text{COD removal rate}=\left(\text{CO}{\text{D}}_{\text{influent}}-\text{CO}{\text{D}}_{\text{effluent}}\right)/\text{CO}{\text{D}}_{\text{influent}}{\;}^{*}\;100 \end{array}$$

Samples were recuperated from anolytes of each reactor, and the COD was measured using LCK 514 (100–2,000 mg/O_2_) after a 1/6 dilution and filtration through the syringe of the chloride elimination kit LCW925 (Hach Lange) to remove any analytical interference from the chloride ions in the media.

### Epifluorescence Microscopy

To study global biofilm structure at the end of experiments, WEs covered by HSCE and/or TDWW microorganisms were removed from the reactors and immediately stained with acridine orange 0.01% (A6014 Sigma) for 10 min. Then, bioanode coupons were washed carefully with sterile physiological water after incubation and dried at ambient temperature overnight. Biofilms were then imaged with epifluorescence microscopy as described by Blanchet et al. (2016), Rousseau et al. (2016), and Askri et al. (2019).

### Microbial Diversity Analysis of HSCE and TDWW Samples and Biofilms

Genomic DNA extractions from HSCE, TDWW, and biofilms were performed using a NucleoSpin® Soil kit according to the manufacturer's instructions. The purity [absorbance ratio (A_260_/A_280_)] and DNA concentration measurements (ng μl^−1^) were checked by Nanodrop. Then, Illumina Miseq 16S rRNA sequencing was performed in order to analyze the composition of the microbial communities in HSCE and TDWW samples and in different biofilms. The 16S rRNA gene V4 variable region PCR primers 515/806 were used in single-step 30 cycles PCR using the HotStarTaqPlus Master Mix Kit (Qiagen, USA). Sequencing was performed at MR DNA (www.mrdnalab.com; Shallowater, TX, USA).

### Statistical Analyses

All experiments were performed in triplicates under the same conditions to prove the reproducibility of experimental results. The mentioned results are the average values with a standard deviation.

## Results and Discussion

### Bioanode Growth and Electrochemical Characterization

Four microbial bioanodes were independently grown by chronoamperometry with a VSP multichannel potentiostat (Biologic SAS), setting the potential value of the WEs at −0.1 V/SCE. This electrode potential has particularly shown its relevance to efficiently lead to the formation of particularly robust and efficient bioanodes from wastewater (Blanchet et al., 2015) and also from sediments (Erable et al., 2017).

**Figure 1:**
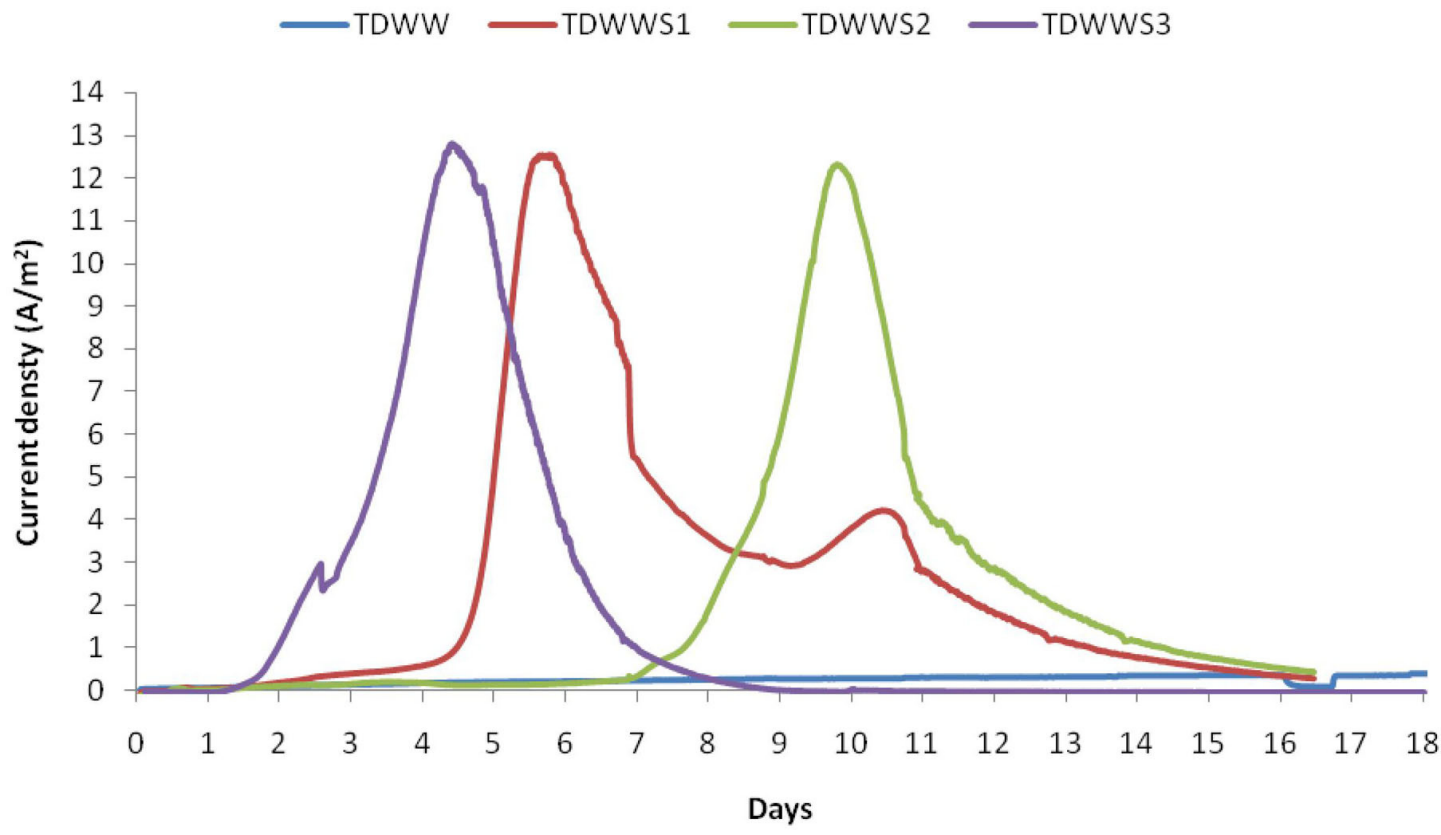
Evolution of the current density (A/m^2^) vs. time (days) for experiments on a carbon felt electrode of 6 cm^2^ projected surface area polarized at −0.1 V/SCE in a reactor containing textile dyeing wastewater (TDWW) and reactors containing 80% of TDWW and 20% of saline sediments (TDWWS1, TDWWS2, TDWWS3).

The four bioanodes grown at this polarization potential are referred to as TDWW, TDWWS1, TDWWS2, and TDWWS3 all through the figures and text. The constant electric polarization was maintained for 17 days, which is a reasonable amount of time to obtain matured bioanode on carbon based electrodes colonized by halo-exoelectrogenic bacteria (Erable et al., 2009; Rousseau et al., 2014; González-Muñoz et al., 2018; Askri et al., 2019). Current densities vs. time plots for −0.1 V/SCE polarization potential are shown in Figure 1. The start of current production was observed almost after 2 days of polarization for all three bioanodes co-inoculated with HSCE, in comparison with the TDWW bioanode, where current production was only recorded from day 5 with very low densities throughout the experiment. This suggests that the electroactive community developed faster and more efficiently in reactors inoculated with HSCE. Remarkably, the maximal current performance reached during the experiments was almost the same for the three bioanodes TDWWS1, TDWWS2, and TDWWS3. A reproducible current production of about 12.5 ± 0.2 A/m^2^ was obtained. However, maximum current density peaks were reached after different periods of polarization for 4, 6, and 10 days for TDWWS3, TDWWS1, and TDWWS2, respectively. Successive batches of addition of textile wastewater usually make it possible to harmonize the current densities over the long term of several replicates of experiments. Here, the reproducibility of the TDWWS replicates observed from the first batch is to be underlined and certainly comes from the inexistence of competitive reactions due to the inhibition of non-electroactive microorganisms by the high salinity, the toxicity of the pollutants, and the possible presence of oxygen traces.

Previous works have shown that the performance of bioanode systems is basically inversely proportional to the complexity of the substrate as in the case of TDWW (Pant et al., 2010; Pandey et al., 2016; Heidrich et al., 2018). However, it is worth noting that in this study, the oxidation currents obtained are almost doubled compared with those obtained with the same inoculum (HSCE) using lactate (5 g/L) as substrate (Askri et al., 2019), and the initial COD for both sets of experiments was 975 and 1,432 mg/l, respectively. The diversity of the TDWWS biofilm composed jointly of autochthonous and allochthonous bacteria may be critical to its performance.

Overall, wastewater used to feed BES, mainly MECs and MFCs, was either domestic wastewater or industrial wastewater from a wide variety of sources (breweries, dairies, refineries). The highest current densities obtained by BES fed with real industrial effluents were 10.7 and 10.3 A/m^2^ where biorefinery (Pannell et al., 2016) and brewery wastewaters (Yu et al., 2015) have been used, respectively. However, for BES fed with domestic wastewater, the highest current densities obtained were 3.8 (Ullery and Logan, 2015) and 3.5 A/m^2^ (Blanchet et al., 2016). The average current densities calculated from 48 research papers are 2.6 A/m^2^ for industrial wastewater and 0.8 A/m^2^ for domestic wastewater. Two main reasons could explain in part this significant difference in average current densities: (i) industrial wastewater is generally more conductive (7 mS/cm) than domestic wastewater (1.5 mS/cm) (Yen et al., 2016) and (ii) the total organic matter concentration in industrial wastewater is between 5,000 and 12,000 mg/L (Rajeshwari et al., 2000), whereas that in domestic wastewater is between 320 and 740 mg/L (Almeida et al., 1999).

The obtained results demonstrated that the TDWW, although rather recalcitrant to biological treatment, is found as the most suitable effluent to generate electric current using hypersaline sediment as a source of tenacious and exoelectrogen biocatalysts.

**Figure 2:**
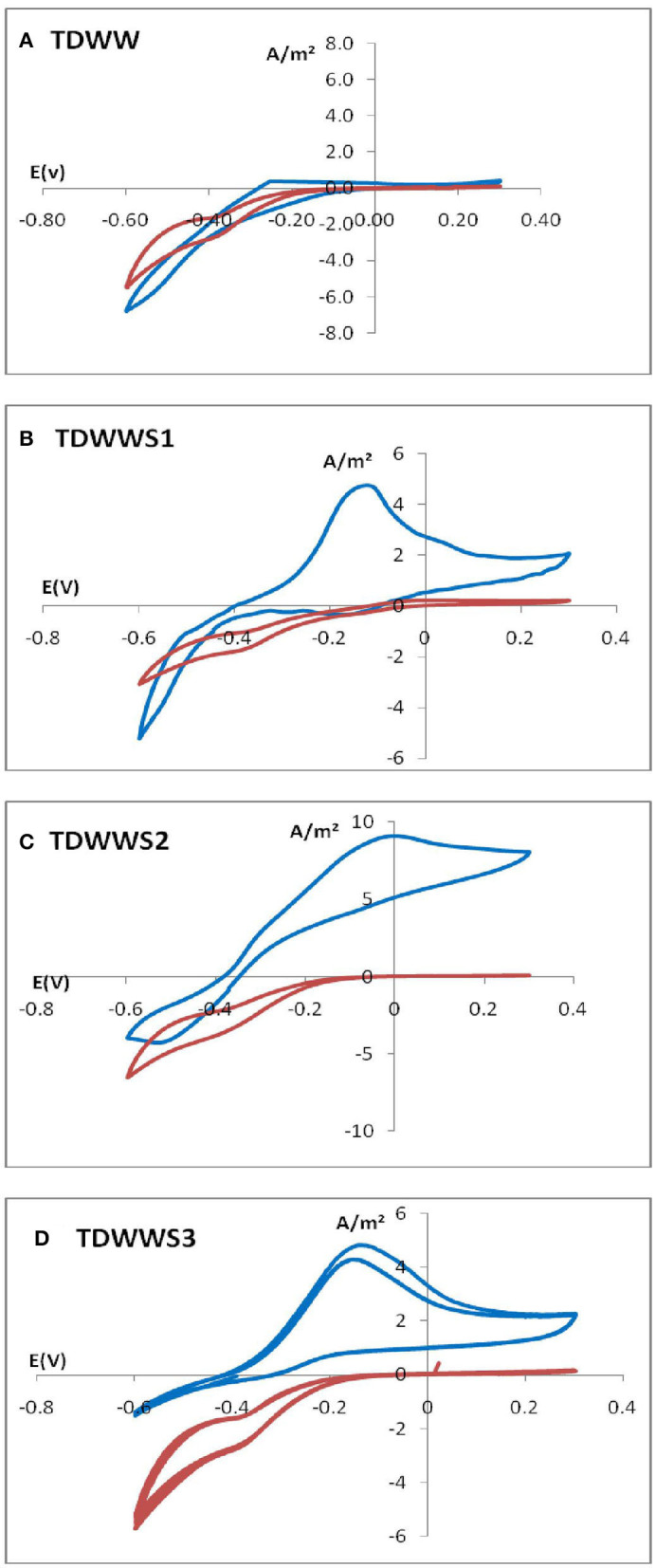
Cyclic voltammetry performed in different conditions: **(A)** CV of porous carbon felt electrode immersed in TDWW; **(B–D)** CV of porous carbon felt electrode immersed in TDWW and HSCE sediment obtained at two different times: CV_I_ (–): at the beginning of the experiment; CV_M_ (–) at maximum current density.

CV was used as a tool to confirm the presence of electroactive biofilm on the electrode surface. Results from CV tests for the four different bioanodes are shown in Figure 2. The initial CVs of the TDWW (negative control) and TDWWS1, S2, and S3 on the porous carbon felt electrode clearly showed the absence of an electroactive biofilm due to the fate shape of the voltammogram from 0.0 to +0.3 V. Interestingly from 0.0 to −0.6, a sharp decrease of the current was observed very likely due to the electrochemical reduction of some compounds in the medium. Another CV cycles were performed when the maximum values of the current density were reached for all reactors (turnover CVs). For the TDWW, no noticeable change from its initial state is visible, confirming the total absence of development of an electroactive biofilm on the corresponding carbon felt electrode. On the other hand, for the other samples, TDWWS1, S2, and S3, a remarkable difference in the general shape of the turnover CVs is identified compared with their initial states and with the TDWW.

All three TDWWs turnover CVs have a zero current potential very close to −0.4 V/SCE. Below this potential, a reduction current is detected that increases to the lower potential limit of −0.6 V/SCE. This reduction current is a less pronounced residual of the same reduction phenomenon(s) observed on all electrodes at t = 0.

Concerning the visible oxidation current for potentials higher than −0.4 V/SCE, it is constantly increasing from −0.4 to −0.1 V or even 0.0 V/SCE. Beyond these potential values, i.e., for potentials more positive than −0.1 V or 0.0 V/SCE, the maximum speed of the exchange current is reached, and a current plateau is therefore observable between −0.1 V and +0.3 V/SCE. The current density even tends to decrease over this anode potential range in the case of Figures 2B,D because the scanning speed is certainly too fast to ensure a steady state of bacterial metabolic phenomena limiting the production of the electron flow.

In sum, the CV results clearly indicate the establishment of a biofilm with electrocatalytic properties on the carbon felt electrodes, as well as a successful enrichment of this electroactive biofilm with bacteria capable of using electron acceptor electrodes (Harnisch and Freguia, 2012; Rimboud et al., 2014). The hysteresis identified between the forward and return curves of the CVS (Figures 2B–D) is classic of bioanodes formed in saline or hypersaline environments (Erable and Bergel, 2009; Rousseau et al., 2014), where capacitive phenomena related to the high ionic charge of the electrolyte and especially the capacity of the electrode material and electroactive biofilm couple coexist.

### COD Measurement

Table 1 indicates that reactors inoculated with HSCE showed satisfactory performance in terms of both current production and COD removal that were about 12.5 ± 0.2 A/m^2^ and 91 ± 3%, respectively. Interestingly, these reactors demonstrated COD removal proportional to the production of current generation. However, COD removal in the reactor not inoculated with HSCE was much lower, i.e., 42.3%, than those in the inoculated reactors, and the production of the current also remains very low. For bioremediation of wastewater containing recalcitrant pollutants, these findings revealed that reactors inoculated with HSCE were found to be comparatively higher in COD removal efficiency than other textile wastewater treatments, such as (i) anaerobic internal circulation reactor (COD removal = 87%), (ii) Fenton's process with and without pH adjustment where COD removal was 89 and 33%, respectively (Hayat et al., 2015), and (iii) the combination of homogenization decantation and membrane treatments (COD removal = 66%) (Buscio et al., 2015).

**Table 1:** COD removal efficiency of TDWW from different reactors.

**Reactors**	**Contents**	**Temperature (^**°**^C)**	**Duration (day)**	**Maximal current density (A/m^**2**^)**	**COD removal rate (%)**
1	20% HSCE + 80% TDWW	45	17	12.5	91.0
2	20% HSCE + 80% TDWW	45	17	12.3	93.6
3	20% HSCE + 80% TDWW	45	17	12.7	88.3
4 (negative control)	100% TDWW	45	17	0.3	42.3

### Microscopy Analysis of Biofilms Morphology

**Figure 3:**
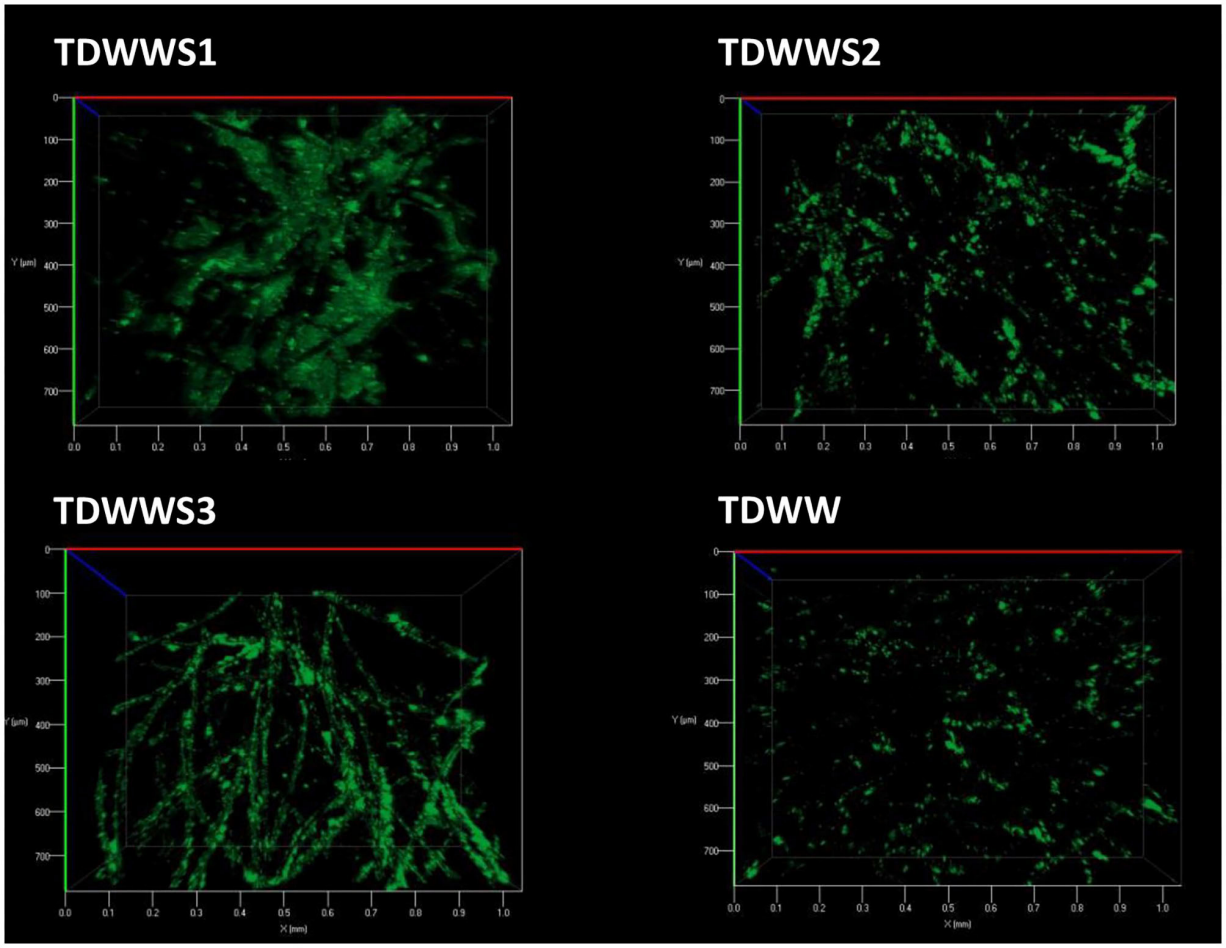
Evaluation of microbial proliferation on the outer surface of carbon felt electrodes by epifluorescence microscopy imaging. Carbon felt electrode surface has been stained with acridine orange to localize living and dead microbial cells.

Colonization of the carbon felt electrodes was evaluated at the end of the experiments, mainly on the outer surface of the felts but also within the porosity of the felt. Indeed, previous work has shown the difficulty of electroactive biofilms to colonize the internal surfaces of felt electrode structures (Chong et al., 2019), especially when real effluents either viscous, highly charged with suspended solids, or very highly loaded with COD are used (Blanchet et al., 2016). Carbon felt WEs possibly covered with HSCE and/or TDWW microorganisms were removed from the reactors and imaged by epifluorescence microscopy (Figure 3). Samples of carbon felt WEs were TDWW that is a bioanode with very low current production (300 mA/m^2^) and TDWWS1, TDWWS2, and TDWWS3 that produced similar current densities of 12.5 ± 0.2 A/m^2^.

TDWW was the electrode sample with the lowest surface colonization rate. Only a few fibers on the surface of the carbon felt electrode were partially colonized by scattered, non-continuous, sparse groups of bacterial colonies with low bacterial cell density. Since this bioanode generated almost no current, it is not surprising to observe a low rate of colonization. The few isolated clusters of bacterial colonies are probably mostly similar to suspended biological substances, kinds of microbial flocs participating in the non-bioelectrochemical oxidation of the COD of textile effluents.

In comparison, the electrode surfaces of TDWWS1, TDWWS2, and TDWWS3 were much more thickly colonized. In spite of relatively similar bioelectrochemical behaviors, but with a time lag, the microscopic patterns of the biofilms show significant differences. TDWWS1 was colonized by a continuous biofilm settled between the surface fibers. The production of exopolymeric substances was also much more prominent for this biofilm. TDWWS2 showed a non-homogeneous colonization of the carbon fibers. The appearance of the biofilm was like the control not inoculated with sediment from HSCE, but the density and number of microbial clusters were much higher. Finally, TDWWS3 showed an intermediate colonization profile between TDWWS1 and TDWWS2, with a thin continuous biofilm that closely covered the surface fibers of the carbon felt. This type of electroactive biofilm enveloping the carbon fibers is generally a feature of biofilms in hypersaline environments (Rousseau et al., 2014). However, we have also recently revealed that the physical structure of electroactive biofilms formed from sediments from HSCE, i.e., enveloping the fibers or distributed between the fibers, was not systematically a sign of improved or, on the contrary, decreased current production (Askri et al., 2019).

**Figure 4:**
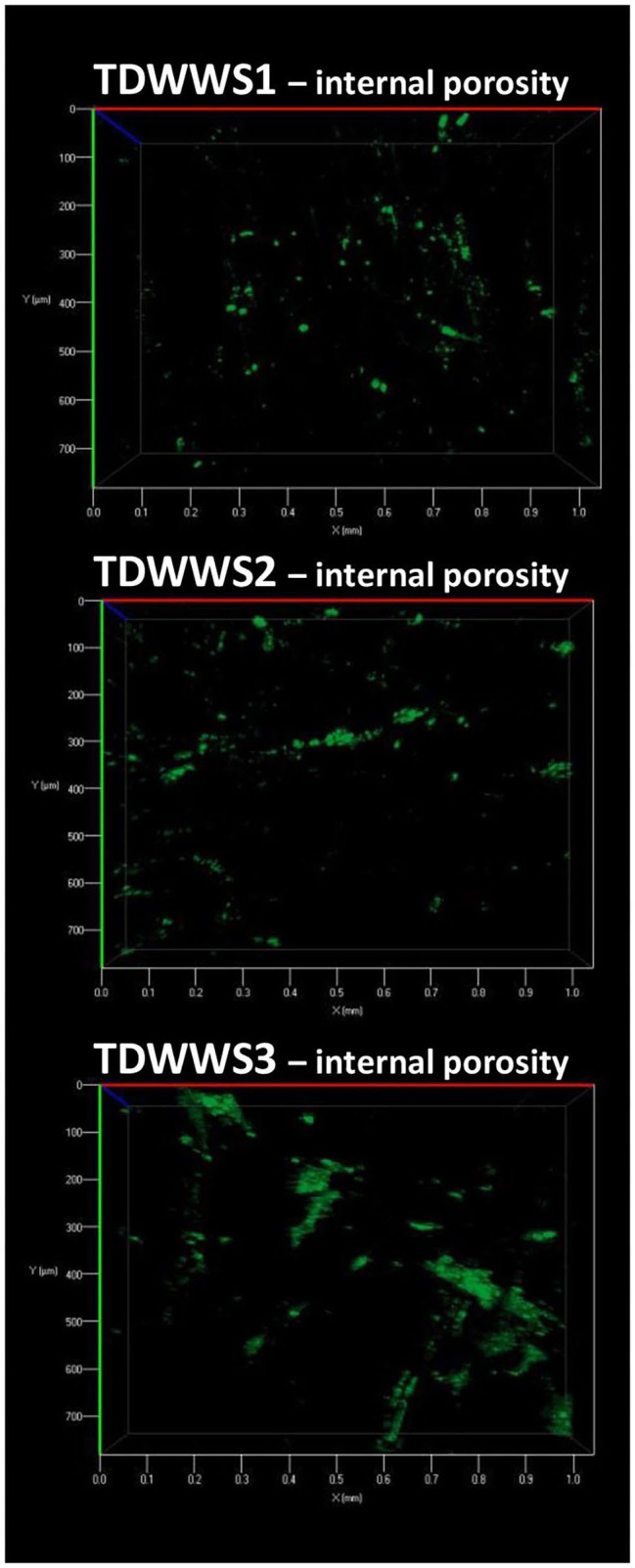
Observation of the internal microbial colonization of the carbon felt electrodes. A 1 cm thick cross-section of the electrodes was prepared, and observations were then performed in the center of the porosity of the electrodes after staining the dead and living microbial cells with acridine orange.

Microscopic inspection of the internal porosity of the carbon felt electrodes revealed a very low degree of internal porosity colonization for the electrodes TDWWS1, TDWWS2, and TDWWS3 (Figure 4). Even so, no trace of biofilm could be seen in the core of the TDWW electrode belonging to the electrochemical reactor not inoculated with HSCE. The poor accessibility to the porosity of the carbon felt is usually due to the clogging phenomena of the external pores of the electrode exerted by particles in suspension present in the wastewater (Blanchet et al., 2016). Insofar as the external faces of the carbon felts do not present a colonization obstructing the pores, i.e., the inter-fiber spaces, this hypothesis is not at all conceivable here. The lack of agitation of the reaction medium, the hydrophobicity of the carbon felt, and the low growth rate of microorganisms in the textile wastewater are certainly individual or combined tracks that can partly justify the low internal colonization rate of the 3D felt electrodes.

### Molecular Analysis of the Bacterial Communities

**Figure 5:**
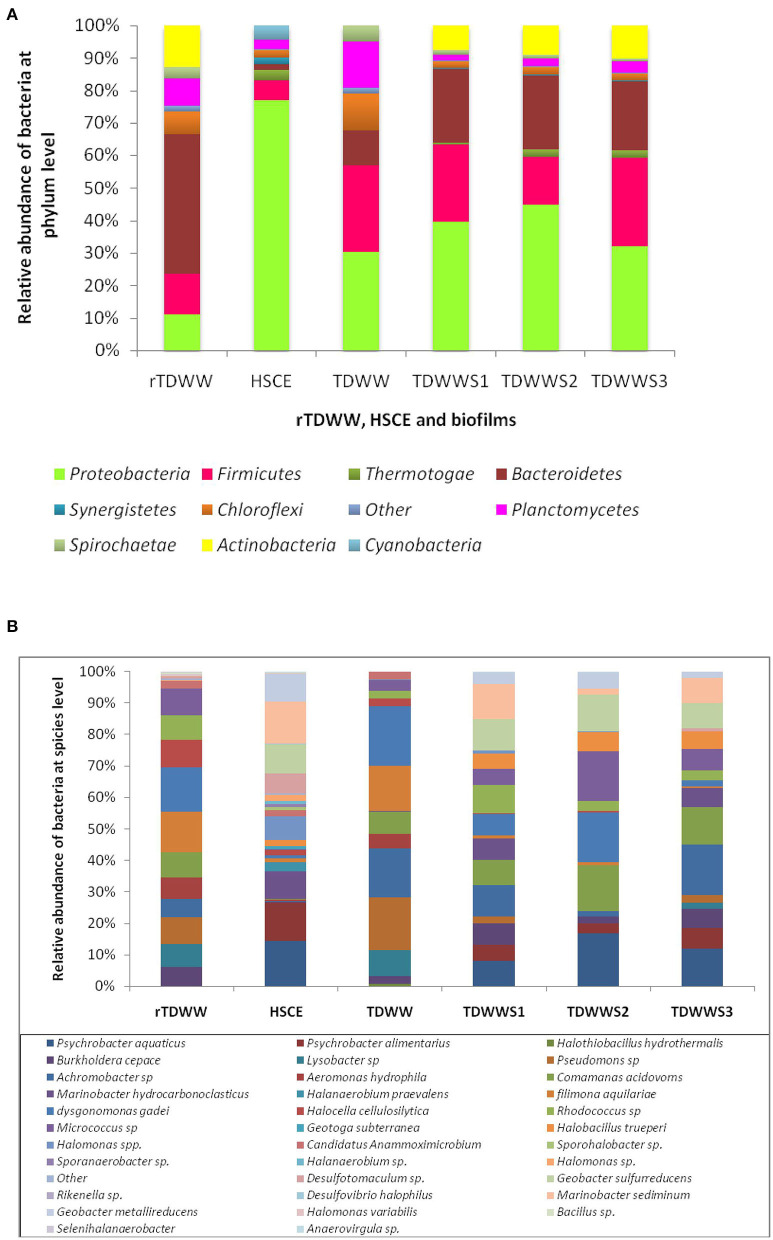
**(A)** Bacterial distribution at phylum level of samples from raw textile dyeing wastewater (rTDWW), hypersaline sediment of Chott El Djerid (HSCE), and biofilms from a reactor containing textile dyeing wastewater (TDWW) and reactors containing 80% of TDWW and 20% of saline sediments (TDWWS1, TDWWS2, and TDWWS3). **(B)** Bacterial distribution at species level of samples from rTDWW, HSCE, and biofilms from a reactor containing TDWW and reactors containing 80% of TDWW and 20% of saline sediments (TDWWS1, TDWWS2, and TDWWS3).

The composition and abundance distribution of each sample (rTDWW and HSCE) and biofilms (TDWW, TDWWS1, TDWWS2, and TDWWS3) at the taxonomic levels of phylum and species are shown in Figures 5A,B.

At phylum level (Figure 5A), raw textile dyeing wastewater (rTDWW) bacterial community, considered as the autochtone community, was mainly characterized by Bacteroidetes (48.5%), Actinobacteria (14.5%), Proteobacteria (12.5%), Planctomycetes (9.5%), and Chloroflexi (8%). Previous studies have demonstrated that the bacterial phyla Acidobacteria, Planctomycetes, and Chloroflexi were more abundant in samples from textile wastewater (Meerbergen et al., 2016). However, in hypersaline sediment sample of Chott el Djerid (HSCE), source of the allochthonous community, the most abundant phylum was Proteobacteria (86%) approximately seven times higher than that in rTDWW (12.5%). In addition to Proteobacteria, the HSCE sample was hosted by other low-grade phyla, such as Firmicutes (6.8%), Cyanobacteria (5%), and Bacteroidetes (2%). Earlier studies using different molecular methods (Ben Abdallah et al., 2016, 2018) have demonstrated that the bacterial community in HSCE was dominated by Proteobacteria, followed by Firmicutes, Bacteroidetes, Cyanobacteria, and Actinobacteria. Based on the species assignment results (Figure 5B), *Dysgonomonas gadei* and *Filimana aquilariae* were the two dominant species in rTDWW, wherein their relative abundances were 21 and 19%, respectively. From the detected phyla Actinobacteria, *Micrococcus* sp. and *Rhodococcus* sp. were the most representative species at relative abundances of 12.6 and 11.5%, respectively. However, the Proteobacteria phylum shows a bacterial profile with the enrichment of *Pseudomonas* sp. (12.5%), *Comamonas acidovorans* (12%), *Lysobacter* sp. (11%), *Aeromonas hydrophila* (10.2%), *Burkholderia cepacia* (9%), and *Achromobacter* sp. (8.5%). By contrast, sample coming from HSCE was represented by various species of Proteobacteria with a predominance of *Psychrobacter aquaticus* (14.5%), *Marinobacter sediminum* (13.5%), *Psychrobacter alimentarius* (12.5%), *Geobacter sulfurreducens* (9.5%), *Marinobacter hydrocarbonoclasticus* (9%), *Geobacter metallireducens* (9%), and *Halomonas* spp. (7.5%).

Figure 5B indicates that the relative abundances of bacteria at phylum level in TDWWS1, TDWWS2, and TDWWS3 biofilms are quite similar, whereas those in TDWW biofilm show some differences. TDWWS1, TDWWS2, and TDWWS3 biofilms demonstrated the presence of the same phyla and largely at the same pattern of abundance Proteobacteria > Bacteroidetes = Firmicutes > Actinobacteria > Chloroflexi = Planctomycetes > Thermotogae. As example, TDWWS1 biofilm showed Proteobacteria (30.5%) > Bacteroidetes (17.43%) = Firmicutes (17.5%) > Actinobacteria (5.8%) > Chloroflexi (1.5%) = Planctomycetes (1.6%) > Thermotogae (0.35%). It is worth noting that the bacterial phyla identified in the three biofilms show heterogeneous profiles composed of phyla found in rTDWW and in HSCE. In this case, TDWWS1, TDWWS2, and TDWWS3 biofilms harbored autochthonous phyla from rTDWW represented in particular by Bacteroidetes, Actinobacteria, Chloroflexi, and Planctomycetes and allochthonous phyla from HSCE, such as Proteobacteria, Firmicutes, and Thermotogae. The heterogeneous bacterial profile of the different biofilms occurs more clearly at species level. *P. aquaticus* (8.06–16.02%), *P. alimentarius* (3.04–6.5%), *G. sulfurreducens* (8.2–11.8%), *G. metallireducens* (2.5–5.4%), *M. hydrocarbonoclasticus* (6.3–7.4%), and *M. sediminum* (2.3–11.7%) as allochthonous species (and not found in rTDWW sample) were the most abundant species in TDWWS1, TDWWS2, and TDWWS3 biofilms. Furthermore, *B. cepacia* (2.4–7.6%), *Achromobacter* sp. (10.1–16.4%), *C. acidovorans* (8.4–14.2), *D. gadei* (2.6–15.0%), *Rhodococcus* sp. (3.2–9.6%), and *Micrococcus* sp. (5.8–15.7%) as autochthonous species (and not found in HSCE) were also abundant in TDWWS1, TDWWS2, and TDWWS3 biofilms.

By comparing the bacterial community of these three biofilms to that hosted TDWW, considered as a control, we found that TDWW harbored less bacteria belonging to the Proteobacteria (9.47%) and Bacteroidetes (3.22%) phyla. However, it hosted more bacteria from the Planctomycetes (4.5%), Chloroflexi (3.6%), and Spirochaeta (1.5%) phyla. At species level, the most abundant bacteria in TDWW biofilm were *D. gadei* (19.04%), *Pseudomonas* sp. (16.66%), *Achromobacter* sp. (15.47%), *F. aquilariae* (14.28%), and *Lysobacter* sp. (8.33%). This biofilm (TDWW) composed only of autochthonous bacteria was not electrochemically effective, as shown in Figure 1. Only few hundred milliamperes were obtained as current generation (300 mA). These strains were also noticed in previous studies on microbial communities in textile wastewater (Meerbergen et al., 2016).

Strikingly, high current production was obtained (12.5 ± 0.2 A/m^2^) with biofilms (TDWWS1, TDWWS2, and TDWWS3) incorporated by both bacteria from HSCE and TDWW samples. A core bacterial community that was shared by the three biofilms could be highly involved in the current production. This core was composed of three autochthonous strains, *Achromobacter* sp., *C. acidovorans, D. gadei*, and four allochthonous strains, *P. aquaticus, G. sulfurreducens, G. metallireducens*, and *M. sediminum*.

## Conclusion

This work is the first to demonstrate the potential to develop a novel halothermotolerant bioanode incorporating allochthonous and autochthonous bacteria from both hypersaline sediment and TDWW. A core bacterial community composed of three autochthonous strains, *Achromobacter* sp., *C. acidovorans, D. gadei*, and four allochthonous strains, *P. aquaticus, G. sulfurreducens, G. metallireducens*, and *M. sediminum*, ensures the effectiveness of the bioanode by producing high current density (12.5 A/m^2^) and a total of 91% of COD removal efficiency. These findings, achieved under both hypersaline (165 g/L) and thermophilic conditions (45°), could lead to possible applications of BES technology for treatment and energy recovering from high-temperature and high-saline wastewaters.

## Data Availability Statement

The original contributions presented in the study are publicly available. This data can be found in Zenodo: http://doi.org/10.5281/zenodo.4276276.

## Author Contributions

HC, BE, RA, and AC: conceived and designed the experiments and analyzed the data. RA, SS, MN, and HC: sampling. RA, BE, LE, and HC: performed bioelectrochemical experiments. BE, RA, and HC: performed electrochemical analysis. HC, BE, RA, SS, MN, and AC: performed microbial and genomic analyses. HC, RA, BE, and AC: manuscript preparation and revision. HC, BE, and AC: supervised the entire project. All authors: contributed to the article and approved the submitted version.

## Conflict of Interest

The authors declare that the research was conducted in the absence of any commercial or financial relationships that could be construed as a potential conflict of interest.
